# Person-Centered Impact of Adult Day Services for People Living With Dementia and Their Caregivers

**DOI:** 10.1093/geroni/igaf122.1708

**Published:** 2025-12-31

**Authors:** Clara Scher, Tina Sadarangani, Shahrzad Siamdoust, Keith Anderson, William Zagorski

**Affiliations:** Weill Cornell Medicine, New York, New York, United States; New York University, New York, New York, United States; New York University, New York, New York, United States; University of Mississippi, Oxford, Mississippi, United States; American Senior Care Centers, Inc., Nashville, Tennessee, United States

## Abstract

Researchers and advocates have recognized the untapped potential of adult day services (ADS) for providing person-centered care for people living with dementia (PLWD) and their caregivers. Yet, valid and reliable outcome measures that reflect PLWD’s values, preferences, and strengths are needed to advance our understanding of how ADS can enhance quality-of-life for this population. This study sought to address this gap by exploring the quality-of-life domains most impacted by ADS from the perspectives of PLWD and their caregivers. Six focus groups were conducted at five ADS sites with 57 total participants (51 PLWD and 6 caregivers). Located across the United States (Tennessee, New York, California), the ADS sites served a diverse population of PLWD and their caregivers, such as by racialized identity (e.g., Black, Hispanic, Chinese) and socioeconomic status (e.g., low income). Reflexive thematic analysis of focus group transcripts was guided by Kitwood’s (1997) conceptual approach to cultivating personhood in dementia care. Results revealed that PLWD and their caregivers perceive ADS as improving quality-of-life by fostering inclusion (e.g., relationships with ADS members and staff), attachment (e.g., feeling safe at the ADS), comfort (e.g., trusting ADS members and staff), occupation (e.g., a sense of purpose by attending the ADS), and identity (e.g., promotion of personhood and culture). Findings have implications for developing person-centered outcome measures to systematically evaluate the impact of ADS on quality-of-life outcomes for PLWD and their caregivers living across the United States and beyond.

